# Obituary for Prof. “Willi” Schmitz

**DOI:** 10.1007/s00210-026-05169-0

**Published:** 2026-03-14

**Authors:** Joachim Neumann, Uwe Kirchhefer, Frank Ulrich Müller

**Affiliations:** 1 Institute for Pharmacology and Toxicology, Medical Faculty, Martin Luther University Halle-Wittenberg, Magdeburger Straße 4, 06097 Halle (Saale) Halle (Saale), Germany; 2 Institute for Pharmacology and Toxicology, Medical Faculty, Münster University, Domagkstraße. 12, 48149 Münster, Germany; 3 Office of the Dean, Medical Faculty, Münster University, Albert-Schweitzer-Campus 1, 48149 Münster, Germany


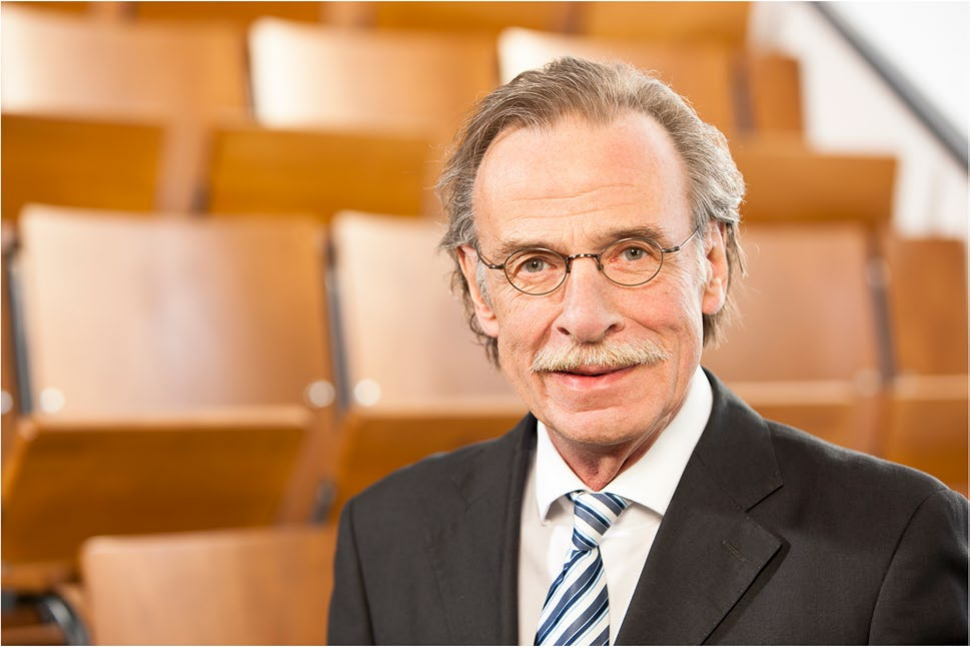
It is with deep sadness and great respect that we commemorate the life and work of Univ.-Prof. Dr. med Dr. h.c. Friedrich Wilhelm Schmitz (or Willi as his friends called him, Fig. 1: credit Foto: Uni MS/Wattendorff). He was born on the 15th of October 1949 in Brühl near Köln (Cologne). He died 170 km away in Münster (Westfalen) on 19th of December 2025. He passed his school final exam (Abitur) in Brühl. He started to study medicine in Lüttich (Liege, Belgium) and then moved to Münster and finally to Mainz. In Mainz, he entered the local Pharmacological Institute. There, he encountered his long-time mentor Prof. Dr. med. Dr. h.c. Hasso Scholz. In his laboratory, he made experiments to study the effect of a cAMP derivative in cardiac preparations (dibutyryl-cAMP) on the force of contraction in animal cardiac preparations for his medical dissertation. This early work laid the foundation for his lifelong scientific focus on cyclic AMP and cardiac signal transduction in health and disease. Then, he joined the lab of Scholz as a post-doc and moved with him to Hannover in 1977 (Erdmann et al. 1981). In this time, in sheep, he raised antibodies against cAMP and cGMP. These tools enabled precise quantification of intracellular second messengers and significantly advanced the understanding of adenosine receptor signaling in cardiac tissue. It turned out that the negative inotropic effects of this receptor in the atrium and ventricle are due to different mechanisms: ion channels, cAMP and phosphatases turned out to be involved.

In 1982, he moved to Hamburg when Prof. Scholz accepted the chair of Pharmacology. Schmitz completed his Habilitation there in 1986 on the mechanism of adenosine in the heart.

At that time, the group was very small, and only another postgraduate electrophysiologist worked on this adenosine project. One of us (JN) started his medical dissertation in Hamburg, as his first medical student in the lab (Neumann et al. 1991). They worked intensively on the role of phosphodiesterase inhibitors and adenosine receptors in the heart (Scholz et al. 1991). When Schmitz moved to Hamburg as assistant to Scholz, he was prepared in a certain way to his later move to Münster. Already in Hamburg, the institute was not using biochemical or radioactive methods. He changed this and worked on cardiac pharmacology using guinea pig, hamster, mouse and rat preparations. He had done contraction experiments in Mainz and Hannover on human ventricular preparations obtained from the local cardiac surgeons in Mainz, Hannover and Hamburg and continued and extended this work. A main interest in the Scholz lab in Hamburg were also cardiac alpha-adrenoceptors. Another topic turned out to be GTP binding-proteins which were an upcoming topic in cardiology at this time (Müller et al. 1993; Eschenhagen et al. 1996). For this topic, another one of us joined his group (FUM). Later, protein kinases and phosphatases were a theme in the lab and here UK entered the group (Glaser et al. 2022). Schmitz became C3- professor for pharmacology in Hamburg in 1988. Then, he started to run for chairmanships in pharmacology in several universities. Eventually, he was offered the chairmanship in Münster and accepted it in 1993. Over the course of several years, he successfully transformed the institute’s infrastructure, establishing modern facilities for molecular biology, radioisotope research, electrophysiology, and transgenic animal models—capacities that had previously been absent. To this end, electrophysiology, calcium transient measurements, protein chemistry and molecular biology methods were instituted that had never existed there before. Several labs were updated for radioactivity and genetic studies. He rapidly became productive in cardiac pharmacology in Münster. This led to the establishment of a cardiac Sonderforschungsbereich in Münster. In later years (2003–2005), he became president of the Deutsche Gesellschaft für klinische und experimentelle Pharmakologie (DGPT). He served as prorector for research in the University Münster (2006–2008). He was chosen to be the first full-time dean of the Medical Faculty in Münster (2008–2016). He was very successful in recruiting new faculty members and helping the faculty to obtain more funding in competitive grants from the local and federal government. For health reasons, he had to step down as dean shortly after being re-elected in an overwhelming way.

The scientific work of Schmitz comprised 213 original papers, 18 reviews, 12 book contributions and about 325 abstracts. This scientific output led to about 10,000 citations. The papers with the highest impact factors were Nature, Lancet (4 times), Circulation (6 times) and Circulation Research (4 times). Over the years, he was mentor to about 35 students of medicine, three students of veterinary medicine, three student of dentistry (3) and to 35 graduate students in other fields. Some of his former students obtained a habilitation (Eschenhagen, Kirchhefer, Linck, Knapp, Neumann, Müller, Stein), a full professorship or a chairmanship in clinical studies (Von der Leyen, Böhm, Jens Scholz) or in pharmacology (Neumann, Müller).

He will be remembered as a good administrator, excellent mentor and a very likable person. It was impossible to have a meeting with him that would not be interrupted by, and did not end with, good-natured jokes. Though he built his academic career far beyond the Rhineland, he never lost the spirit of his origins. Each carnival season drew him back to Cologne, where he immersed himself in the traditions and joyful exuberance of the festivities—a reflection of the humor and lightness he brought to all who worked with him. He will be sadly missed by his family and friends. With his passing, German pharmacology has lost one of its most influential and visionary representatives.
